# Multifunctional metasurface coding for visible vortex beam generation, deflection and focusing

**DOI:** 10.1515/nanoph-2025-0016

**Published:** 2025-03-07

**Authors:** Run Tian, Zhixiao Zhang, Li Gao

**Affiliations:** 12577 Nanjing University of Posts and Telecommunications, School of Science, Nanjing, China; State Key Laboratory of Flexible Electronics (LoFE), Institute of Advanced Materials (IAM), School of Materials Science and Engineering, Nanjing University of Posts and Telecommunications, Nanjing, China

**Keywords:** orbital angular momentum, coding metasurface, multifunction, metalens

## Abstract

Vortex beams, as beams carrying orbital angular momentum (OAM), exhibit unique donut-shaped intensity distributions and helical wavefronts. They are widely applied in fields such as optical communication, nanoparticle manipulation, and quantum information. Traditional vortex beam generation methods, such as those based on Pancharatnam–Berry phase design, can effectively generate vortex beams, but the conversion efficiency and design flexibility are limited by polarization states and incident angles. In addition, the generated and propagated vortex beams require separate metasurface for wavefront deflection and refocusing for practical applications. This work proposes a novel metasurface design approach based on resonant phase, where phase coverage of 2 ***π*** is achieved by varying the radius of the nanocylinders. In addition to the efficient vortex beam generation in the visible regime, we have tackled the challenge of simultaneous control of vortex beam’s anomalous deflection and refocusing, through different encoding sequences superimposed based on the principle of Fourier convolution and metalens design. This all-in-one multifunctional metasurface design offers new technological pathways for secure optical communication and quantum manipulation applications.

## 1 Introduction

Optical vortices refer to a type of beam that carries a helical phase, formed by the spiral rotation of the wavefront around the optical axis [1], [2], [3]. Compared to traditional plane waves and spherical waves, vortex beams exhibit unique characteristics such as a singular phase at the center and a donut-shaped intensity profile. These distinct physical properties have driven their applications in various fields, including optical communication [4], [5], [6], nanoparticle manipulation [7], [8], [9], quantum information [10], [11], [12], and so forth. In the visible and near-infrared (NIR) bands, vortex beams are especially important due to their high spatial coherence, unique phase structures, and the ability to encode information in the orbital angular momentum (OAM) degree of freedom. This opens up new avenues for applications such as OAM holography [13], [14], [15], [16] and polarization detection [17]. In recent years, the trend toward further miniaturization of optical systems has created an urgent demand for ultra-small vortex phase plates. Traditional devices such as spiral phase plates, q-plates, and diffractive optical elements are typically much larger than the working wavelength [18], [19], [20]. However, a series of techniques for generating optical vortices using metasurfaces have recently been developed.

Metasurfaces are two-dimensional arrays composed of subwavelength structures that can shape the wavefront of light with extreme design flexibility. Pioneer studies, such as those by Capasso et al. [21], demonstrated the ability to control light using metasurfaces, generating anomalous diffraction patterns and creating vortex beams by manipulating the phase distribution on the surface. Subsequently, metasurfaces designed based on the Pancharatnam–Berry (PB) phase have been widely used for vortex beam generation and vortex beam focusing [22], [23], [24]. However, this method has certain limitations. PB phase control depends on the polarization state of the incident light and will be generated only for circular cross-polarized waves. Since circularly polarized beams have symmetry, circularly polarized (CP) light is typically used as the incident light to minimize interference [25]. The cross-polarized phase shift depends on the scattering rotation and chirality of circularly polarized waves, which is independent of the incident wavelength. Moreover, under circularly polarized light incidence, the characteristics of PB phase elements are almost insensitive to the incident angle. This significantly limits the metasurface’s sensitivity to wavelength and poses challenges in designing metasurfaces with different transmission properties for varying incident angles [26], [27]. On the other hand, the advantage of resonant phase, which uses linearly polarized incident light, lies in the ability to exploit the degree of freedom in polarization direction. By designing anisotropic structures, one can separately control the transmission and reflection properties in different directions, which is crucial in applications such as beam splitters and polarization separators [28], [29], [30]. Recently, Xiao et al. proposed a novel gradient phase metasurface design [22] aimed at overcoming these limitations. By utilizing phase gradients in the gaps between meta-atoms, they were able to control the local wavefront at the nanoscale. Under the condition of linearly polarized light incidence, this design can generate high-purity optical vortices with different topological charges in the visible spectrum.

While previous work has achieved high-purity vortex beam generation, it becomes quite challenging to simultaneously deflect and focus vortex beams for practical applications, as the generated vortex beam needs to be deflected for different angles and refocused after propagation energy loss. For effective multifunctional metasurface design, Cui has previously introduced the concept of digital coding metasurfaces [31], which simplified the design and optimization process by encoding phase sequences onto meta-atoms. Coding metasurfaces can manipulate electromagnetic waves simply and efficiently, thus by further integrating metasurfaces through convolution operations [32], [33], [34], [35], multifunctional metasurfaces have been realized and demonstrated in the low-frequency range [36], [37]. Similarly, by combining the coding and convolution theory, metasurface can also be designed to achieve simultaneous generation and propagation control of vortex beams in the visible regime.

**Figure 1:**
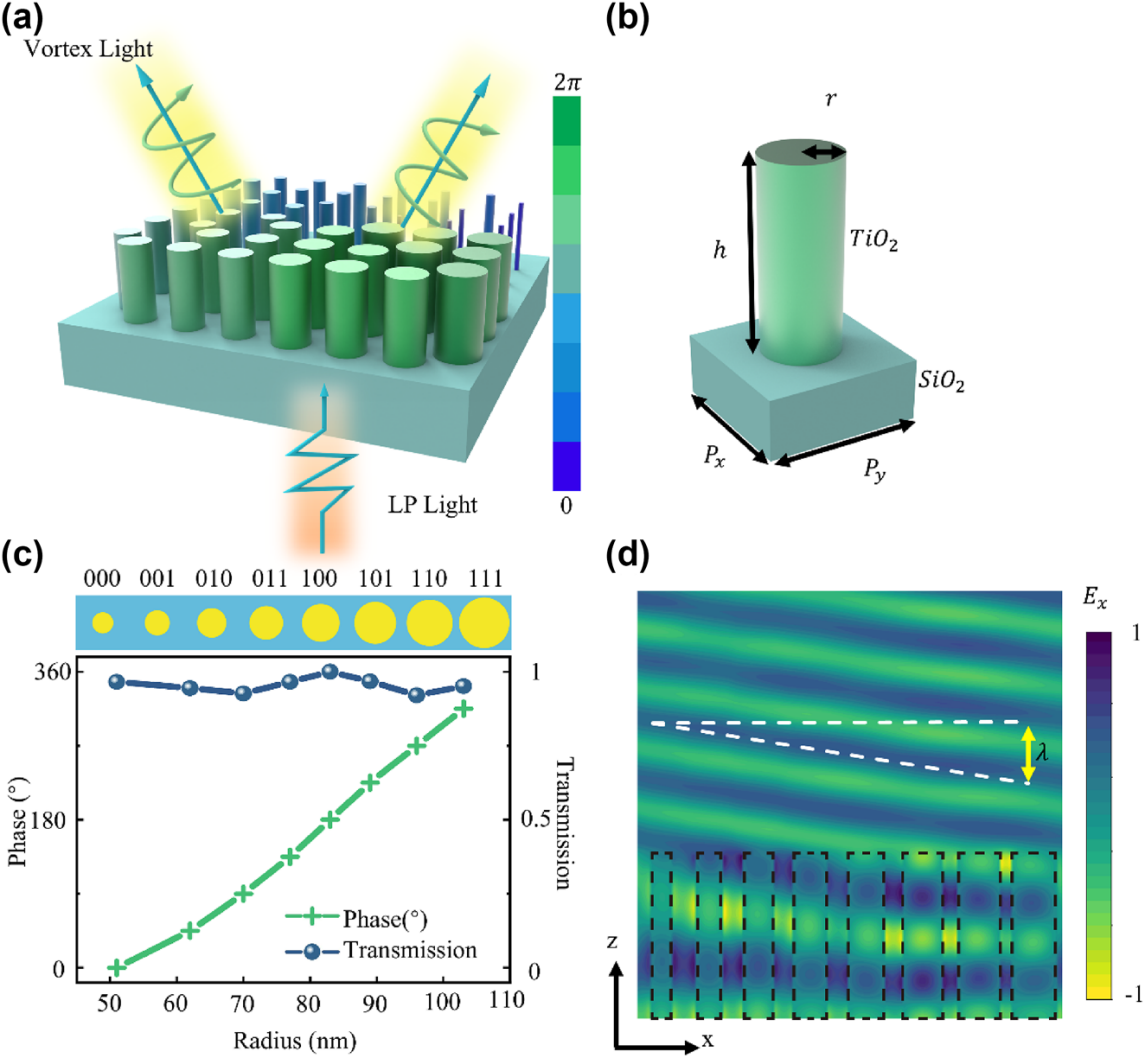
Metasurface array and meta-atom design illustration. (a) Three-dimensional schematic of the multifunctional metasurface. (b) Three-dimensional schematic of the meta-atom unit, *P*_*x*_ = *P*_*y*_ = 250 nm, *h* = 600 nm. (c) Relationship between the phase response and transmittance of the 8 meta-atoms under 532 nm x-linearly polarized light incidence as a function of radius. (d) Electric field distribution of the x-component at 532 nm excited inside the nanocylinder and the transmitted wavefront.

Here, we present a resonant phase encoding metasurface that utilizes a TiO_2_ nanocylinder arrangement to achieve vortex light generation at a wavelength of 532 nm, with the average transmission efficiency of approximately 91 %. By introducing the principle of Fourier convolution from digital signal processing into coding metasurfaces, the flexible control of vortex beam transmission direction can be achieved through the Fourier convolution and summation of different sequences on the coding metasurface. Additionally, by incorporating the principle of coding unit summation in the complex domain, the coding sequences of two functions can be added to realize the superposition characteristics of multifunctional vortex beams, enabling beam splitting of the output vortex light as shown in Figure 1a. Similarly, we combined the vortex beam sequence with the metalens sequence to achieve focused vortex beams, significantly enhancing the intensity at the designed focal length. This all-in-one metasurface approach of generating, deflecting and focusing of vortex beams with significant design flexibility and efficiency holds promise for future applications in high-capacity optical communication and OAM holography.

## 2 Design principle

### 2.1 The meta-atom design for generating vortex beams

Vortex light, also known as vortex waves, is distinguished by its unique helical phase structure. The phase distribution of a vortex beam follows a spiral pattern along its propagation direction, typically represented by an integer *l* known as the topological charge. For example, a “+*l* vortex beam” indicates that its phase contains a helical term *e* ^−*ilθ*^, where *θ* is the azimuthal angle. When the beam rotates around the propagation axis within one period, the phase changes by *l*∗2*π*. Therefore, we arrange the designed coding units within a circular pattern to repeat *l* times, covering the transmission phase range from 0 to 2*π*. Due to the gradient phase delays imposed by the coding units, the transmitted phase exhibits a helical distribution. Theoretically, a two-dimensional helical phase distribution can be expressed as follows:

(1) $$\emptyset \left(x,y\right)=l\theta =l\cdot \arctan\left(\frac{y}{x}\right)$$

Where *x* and *y* represent the coordinates of the meta-atoms along the *x*-axis and *y*-axis in the metasurface.

In most metasurface designs for generating and manipulating vortex light, the abrupt phase shifts are achieved by using PB phase, which involves rotating the same meta-atom at different angles. The PB phase coding metasurface designed by Jing et al. [22] enables flexible modulation of vortex beams, but it requires circularly polarized light as input. This dependence on circular polarization limits the design freedom in terms of polarization direction and adds complexity to the experimental setup which involves additional optical components and alignment.

In our metasurface design, we use TiO_2_ nanocylinders on a silica substrate as the basic coding unit and conducted full-wave simulations using FDTD solutions. By optimizing the parameters of the TiO_2_ nanocylinders and simply adjusting their radius, we can achieve a complete 360° phase shift. This resonant phase design does not require the incident light to be circularly polarized, it can be flexibly manipulated using linearly polarized light. More importantly, the fabrication of nanocylinders with different radius can be easier and more precise compared to rotated meta-atoms with various shapes and angles. Here, we attribute such effect to resonant phase shifts as only the radius change modifies the intensity of each resonant mode, ultimately achieving control over both the phase and amplitude. For high aspect ratio nanocylinders, propagation phase shifts also play a role as phase delay is accumulated when light propagates within the waveguide. In our design, as the height of the nanocylinder waveguide is not changed, we do not discuss propagation phase shifts to simplify the problem.

Due to the presence of multiple excited resonance modes, the analysis becomes challenging. Therefore, we have adopted a high-contrast dielectric waveguide model to explain this phenomenon. According to the waveguide theory of cylinders, the phase shift of the cylinders is related to the effective refractive index *n*_*eff*_, as shown in the following formula.

(2) $$\varphi =\frac{2\pi }{\lambda_{d}}n_{\mathrm{eff}}h$$

The effective refractive index *n*_*eff*_ can be calculated using the waveguide model. Different cylinder diameters correspond to different effective refractive indices, resulting in varying phases.

To cover the whole range $\left(0∼2\pi \right)$ of the phase with a working wavelength of 532 nm, the meta-atom is composed of a TiO_2_ nanoscale cylinder located on glass (SiO_2_) substrate, due to its high refractive index and negligible loss in the whole visible light band. The unit cell is shown in Figure 1b, where *r* represents the radius of the TiO_2_ nanocylinder, *h* represents the height of the TiO_2_ cylinder, *P*_*x*_ and *P*_*y*_ represent the length and width of the SiO_2_ substrate respectively.

To achieve better vortex beam performance, this paper introduces a 3 bit encoding unit, which includes 8 progressive transmission phases specifically designed for vortex beam generation. By using a ring arrangement of 8 encoding units, the x-polarized incident wave can be effectively converted into a vortex beam. So, we optimized the radius parameters of the nanocylinders through scanning their radius by using the FDTD solutions. When the radius varies from 51 nm to 103 nm, it can cover a phase range from 0 to 2*π*. From Figure 1c, the phase difference between adjacent encoding units can be maintained at around 45° near the wavelength of 532 nm. The transmission coefficients of the encoded units are all above 0.9, which meets the basic requirements for efficient transmission encoding metasurface. In the case of three-bit encoding, the entire 360° transmission phase is discretized into eight levels: 0°, 45°, 90°, 135°, 180°, 225°, 270°, and 315°. These levels correspond to the binary representations “000”, “001”, “010”, “011”, “100”, “101”, “110”, and “111”, and are simply represented by numbers 0 to 7, corresponding to the radius of the TiO_2_ nanocylinders as illustrated in Figure 1c. Figure 1d shows the simulated electric field distributions of the transmission waves under excitation at 532 nm. When the nanocylinders are arranged with subwavelength spacing, Fabry–Perot oscillations create resonant standing wave patterns within the cylinder layer. This resonance significantly alters the effective optical path length, resulting in different phase delays *ϕ* in the transmitted wavefront, depending on *r*, as indicated by the varying positions of the dashed lines. Consequently, in this configuration, the cylinder array achieves pure phase control over the wave. By arranging cylinders with gradually increasing *r* within a single unit cell, the x-polarized incident wave can be effectively refracted, as illustrated in Figure 1d.

### 2.2 The metasurface coding for vortex beam deflection

The coding metasurface can manipulate the wavefront of the incident plane wave, thereby generating specific scattering angles. According to the generalized Snell’s law, the angle of deviation of the refracted wave relative to the *z*-axis can be expressed as follows:

(3) $$\theta =sin^{-1}\left(\lambda /\Gamma \right)$$

Where *λ* and Γ represent the wavelength at the working frequency and the period of the coding sequence, respectively.

The Fourier convolution principle is based on the frequency-shift property in Fourier transform theory. It achieves a shift in the scattering pattern by performing a convolution operation between the original coding pattern and a gradient coding sequence. This method allows for flexible and continuous control over electromagnetic wave scattering, enabling the redirection of scattering patterns for various applications, such as beam steering and focusing. At the same time, by extending coding metasurfaces to the complex-number domain, the design leverages the intrinsic nature of electromagnetic waves. The addition theorem of complex codes facilitates multifunctional control, enabling simultaneous operations.

The designed metasurface can manipulate light in different ways at the target wavelength. By combining the functionalities of two metasurfaces, one for beam splitting and one for vortex generation, two different degrees of freedom in light manipulation can be integrated into a single metasurface. Using convolution operations, we can superimpose the coding patterns of the two metasurfaces. Following the method proposed by Cui et al. [34], we derive the shift theorem for metasurface superposition. It is well known that the far-field scattering pattern of a metasurface and the near-field distribution are related through Fourier transforms, as expressed in the following equation:

(4) $$f\left(x_{\lambda }\right)\cdot g\left(x_{\lambda }\right)\overset{\mathrm{FFT}}{\Leftrightarrow }f\left(\sin\theta \right)*g\left(\sin\theta \right)$$

Where *x*_*λ*_ = *x*/*λ* represents the electrical length, and *θ* denotes the angle relative to the normal. Considering the exponential relationship between the field distribution and phase, the phase pattern of the designed metasurface can be obtained just by adding up the phase of the individual structures.

Upon combining the two coding metasurfaces, the transmission phases of corresponding coding units at the same location are superimposed. Furthermore, the interplay of phase superposition can be elucidated as follows:

(5) $$\varphi_{1}+\varphi_{2}\overset{\mathrm{FFT}}{\Leftrightarrow }e^{j\varphi_{1}}*e^{j\varphi_{2}}=e^{j\left(\varphi_{1}+\varphi_{2}\right)}$$

### 2.3 The metalens design for focusing vortex beams

We observed that vortex beams generated by the metasurface always exhibit a divergent nature, with their intensity gradually decreasing in space. To better enable vortex beams for applications in high-capacity optical communications, super-resolution imaging, and optical trapping, we can focus the vortex beam at a designed focal point while maintaining its vortex characteristics. Based on the focusing principle of the optical lens, we usually reshape the incident plane wavefront into a spherical wavefront, the phase mutations at different positions in the radial direction of the metalens is required to satisfy the formula as:

(6) $$\emptyset_{\left(r\right)}=2k\pi +\frac{2\pi \left(\sqrt{f^{2}+r^{2}}-f\right)}{\lambda }$$

Where, *f* represents the theoretical focal length, *r* is the radial distance from the phase discontinuity point to the center of the metalens, and *k* is an arbitrary integer. According to Equation (6), the required phase distribution on the plane of the metalens can be precisely calculated for each discrete unit of the metalens.

We observed that vortex beams generated by metasurfaces always exhibit a divergent nature, with their intensity gradually diminishing in space. To better enable vortex beams for applications such as high-capacity optical communications, super-resolution imaging, and optical trapping, we can focus the vortex beam at a designated focal point while maintaining its vortex characteristics.

## 3 Results and discussion

### 3.1 Vortex beams generation

A vortex beam is a special type of beam characterized by a hollow intensity profile. Under the 532 nm x-polarized light incident, the phase of the optical path of the transmitted beam is altered due to the principle of resonance phase, resulting in the generation of a vortex beam. Vortex beams carry orbital angular momentum. Theoretical studies often focus on the topological charge of vortex beams. When the topological charge *l*=1, a single rotation of the wavefront achieves 360° phase coverage. For a topological charge *l*=2, a 720° phase coverage is achieved through a single rotation of the wavefront plane.

**Figure 2:**
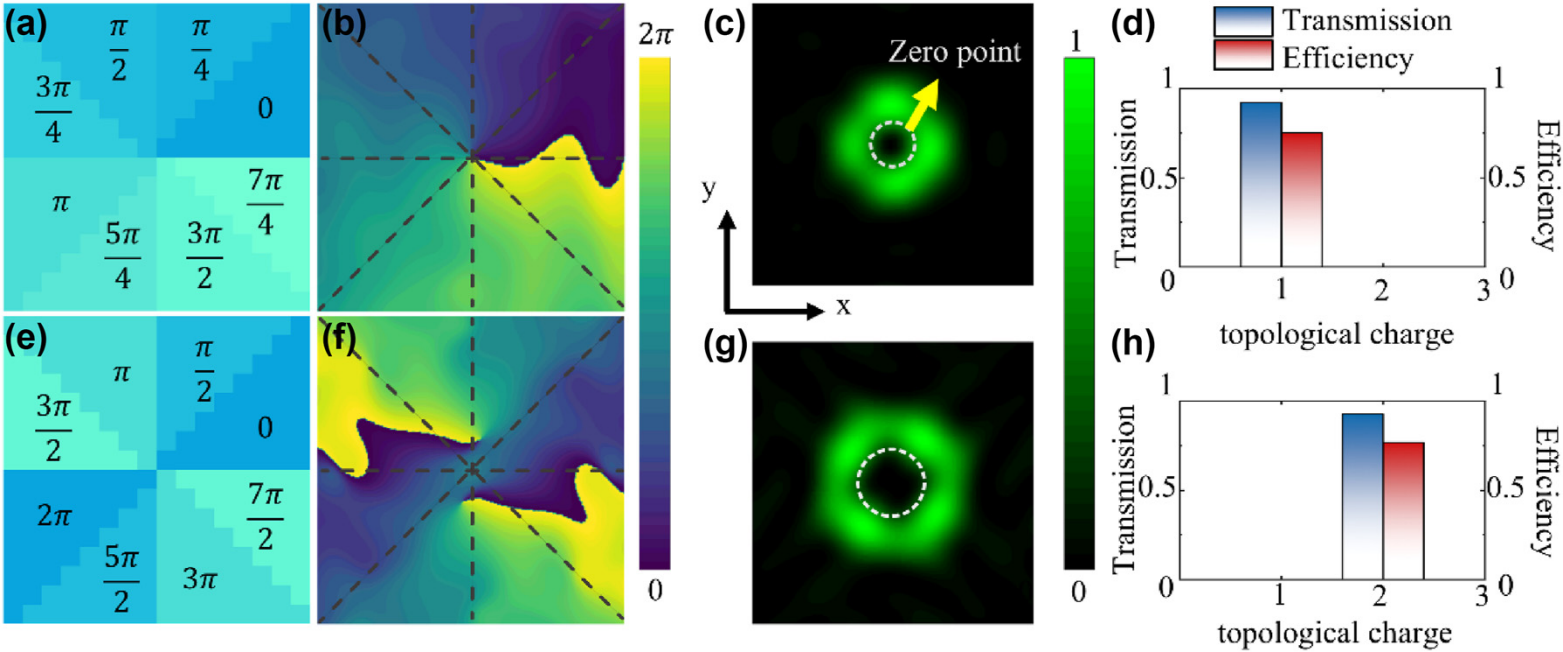
3 bit coding metasurface generating vortex beams with different topological charges. (a) Required phase distributions for generating vortex beams with *L* = 1. (b) Phase distribution and (c) far-field intensity distribution of *L* = 1 vortex Light in numerical simulation, (d) along with the transmittance and conversion efficiency. (e) Required bottom-phase distributions for generating vortex beams with *L* = 2. (f) Phase distribution and (g) far-field intensity distribution of *L* = 2 vortex light in numerical simulation, (h) along with the transmittance and conversion efficiency.

Thus, the helical phase distribution is divided into 8 equal segments, each with a constant phase increment of 45°. This is like a linear phase distribution, and we can encode this helical phase using numbers from 0 to 7. Based on this phase distribution, we designed a 24 × 24 3 bit coding metasurface, where linearly polarized incident light is encoded and transmitted through the metasurface to generate vortex light. The required phase distribution is shown in Figure 2a, and the far-field scattering characteristics of the coding metasurface were numerically simulated by the finite-difference time-domain (FDTD) method. Figure 2b shows that the helical phase change precisely matches a 2*π* variation within one rotation. As shown in Figure 2c, the far-field scattering main lobe clearly exhibits a helical pattern, with the beam intensity at the center of rotation being minimal. The conversion efficiency represents the proportion of the vortex beam intensity relative to the incident light. Figure 2d shows the far-field intensity distribution of the transmitted vortex light, with a transmittance and conversion efficiency of 92.28 % and 75.16 %, respectively.

The amount of angular momentum carried by a vortex beam is determined by its topological charge *l*. Vortex lights with different topological charges exhibit interesting properties, allowing for the customization of their characteristics through the control of the topological charge to suit various applications. Here, we also designed a 24 × 24 coding metasurface for generating vortex light with a topological charge of 2 by modifying the encoding sequence to repeat the phase twice within one cycle from 0 to 2*π*, as shown in Figure 2e. Similarly, using the FDTD method for simulation, Figure 2e shows the phase distribution of the vortex beam, where two 2*π* phase shifts are clearly completed. Figure 2f corresponds to the far-field pattern of the vortex beam, which is a ring-shaped beam with zero intensity at the center. The transmission efficiency and vortex light conversion efficiency are 92.83 % and 76.89 %, respectively, as shown in Figure 2h. The metasurface we designed achieves very low transmission loss and a commendable energy conversion efficiency from linearly polarized light to vortex light.

We have made some comparisons between our designed metasurface and previously reported designs (see Supporting Information Table S1) [38], [39], [40]. Our design of vortex beam generation in the visible spectrum exhibits comparable or superior performance compared to those designs in the long-wavelength range. Specifically, Ref [22] that operates at the same 532 nm wavelength, employs complex hollow nanocylinders optimized through deep learning to achieve higher purity vortex beams but suffered from low transmittance due to high-aspect-ratio structures. Our design maintains good purity and conversion efficiency based on much simpler geometry, and more importantly, achieves multifunctional integration for simultaneous beam deflection and focusing via the convolution theorem.

In addition, we have investigated the applicability of this design to a broader spectral range as shown in Supporting Information Table S2. The results only indicate slight performance decrease at wavelengths of 500 nm, 560 nm and 580 nm. At the same time, manufacturing deviations can impact device performance, thus we have analyzed the effect of fabrication errors, and the results show that the metasurface performance remains robust to such variations (see Supporting Information Table S3).

### 3.2 Vortex beams deflection

We carry out Fourier convolution operation between a coding metasurface and a gradient sequence. According to the angular addition calculation rule, the two coding sequences were combined to produce a metasurface coding sequence capable of deflecting vortex beams. Design and simulation of these two gradient metasurface are summarized in Supporting Information Figure S4.

**Figure 3:**
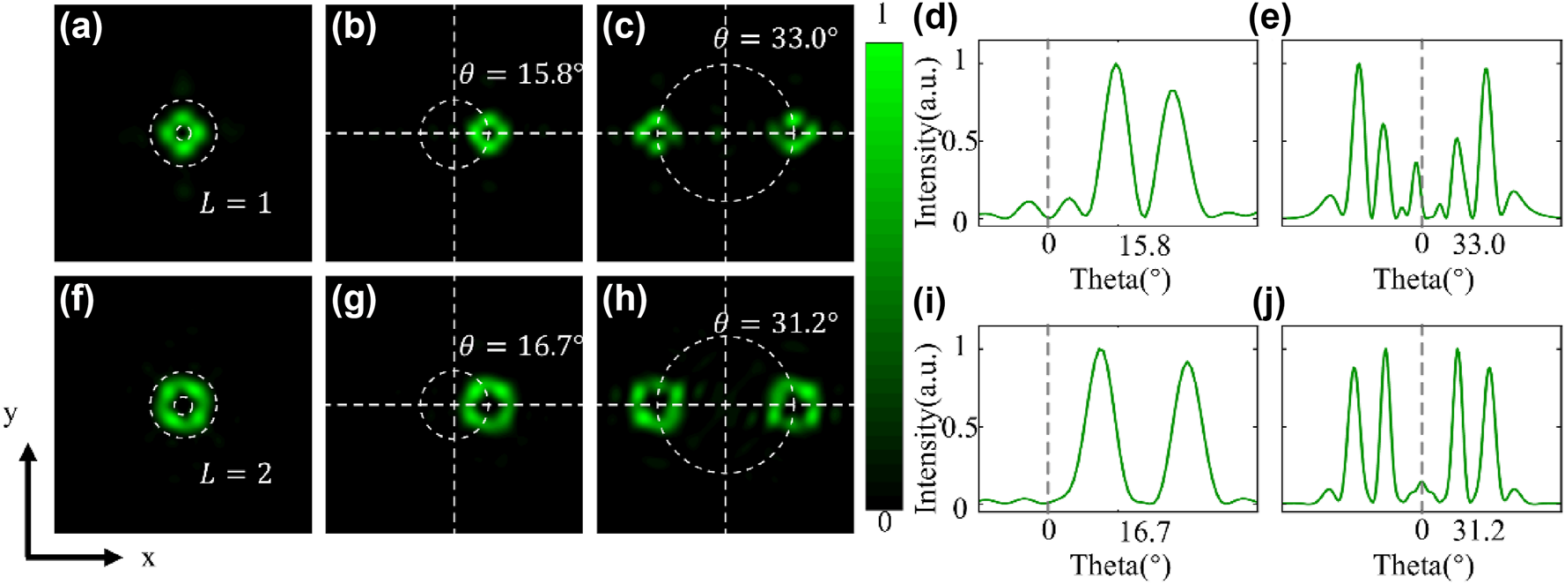
Deflection Characteristics of vortex beams with *L* = 1 and *L* = 2. (a) Far-field intensity distribution of the generated vertical vortex beam with *L* = 1. (b) Far-field intensity distribution of the vortex beam after deflection by superimposing with the “01234567” gradient sequence using the convolution method. (c) Normalized far-field intensity distribution along the horizontal direction (*y*-axis) passing through the center of the vortex beam. (d) Far-field intensity distribution of the deflected double vortex beams obtained by superimposing with the “73377337” gradient sequence. (e) Normalized intensity curve along the horizontal direction passing through the centers of the two vortex beams. Each double vortex beam consists of two main lobes, and the position of the minimum intensity point in the middle represents the deflection angle. (f) Far-field intensity distribution of the generated vertical vortex beam with *L* = 2. (g–j) Summarized far-field intensity and normalized far-field intensity distribution along the horizontal direction of the *L* = 2 vortex beam after superimposing with two gradient sequences, along with the corresponding deflection angles.

As an example of single-beam deflection, after combining two encoded metasurfaces, the transmitted phases of the corresponding encoding units at the same position are superimposed. For instance, when unit “100” (*π*) is combined with unit “101” (5*π*/4), it yields unit “001” (*π* + 5*π*/4=9*π*/4, and 9*π*/4 − 2*π*=*π*/4) using the convolution principle. We designed a 16 × 16 encoded metasurface for vortex beam generation, and its far-field simulation pattern is shown in Figure 3a. When the vortex beam metasurface is combined with the gradient metasurface, deflection of the vortex beam can be achieved, and the far-field simulation result is shown in Figure 3b. Using the convolution principle and adopting a new encoding sequence, we achieved single-vortex beam deflection at a working wavelength of 532 nm. The deflection angle of the vortex beam was 15.8°, and as can be intuitively seen from Figure 3c, the intensity curve exhibits two main lobes, with the minimum intensity at the center, reflecting the unique intensity distribution characteristics of vortex beams. Similarly, for double-beam deflection, by combining the gradient encoding sequence with the vortex encoding sequence, a new encoded metasurface is obtained. This metasurface effectively splits the x-polarized incident wave into a pair of symmetric vortex beams for transmission, with a deflection angle of 33.0°, as shown in Figure 3d. Figure 3f then shows the intensity curve of the deflected vortex beam, highlighting the symmetric characteristics of the double vortex beams. Each double vortex beam consists of two main lobes, with the minimum intensity point located between these two main lobes.

For the vortex beam with topological charge *l* = 2, we also use a 16 × 16 coded metasurface for superposition, and the far-field pattern of the generated vortex light is shown in Figure 3f. According to the convolution principle, by superimposing the vortex metasurface sequence with two gradient sequences, the new metasurface generated can also produce a vortex beam with *l* = 2, and achieve both single-beam deflection and the generation and deflection of a pair of symmetric vortex beams, as shown in Figure 3g–i. The deflection angles are 16.7° and 31.2°, respectively, and the slight discrepancy between the deflection angles and the theoretical values can be optimized by improving the encoding of the second-order vortex beams. Figure 3h–j show the transmitted far-field intensity distribution characteristics, where the intensity zero points are clearly observed at the center of the beams.

### 3.3 Vortex beams focusing

We have further designed a compact metasurface lens array based on 3 bit encoding with a 32 × 32 device size and a total length of 8 μm to verify the focusing characteristics of vortex beams. Once we calculated the phase difference for each unit relative to the center of the focusing metasurface, we used the eight coding units to provide a finer approximation of the required phase at each specific location. For example, when the calculated phase difference is $\Delta \varphi \left(x,y\right)=88^{◦}$ , this phase difference is closest to the encoding unit “010” (90°). Therefore, at that specific location, we fill it with the coding unit “010”.

Based on the phase distribution calculated using Equation (6), we matched the target phase with the 3 bit encoded phase using commercial mathematical software (MATLAB, R2021b). At a working wavelength of 532 nm, we designed focal lengths of *f* = 8 μm, *f* = 10 μm, and *f* = 12 μm, and designed a 3 bit encoded 32×32 small metasurface array with a total size of 8 μm to be combined with the vortex beam metasurface (see Supporting Information Figure S5).

**Figure 4:**
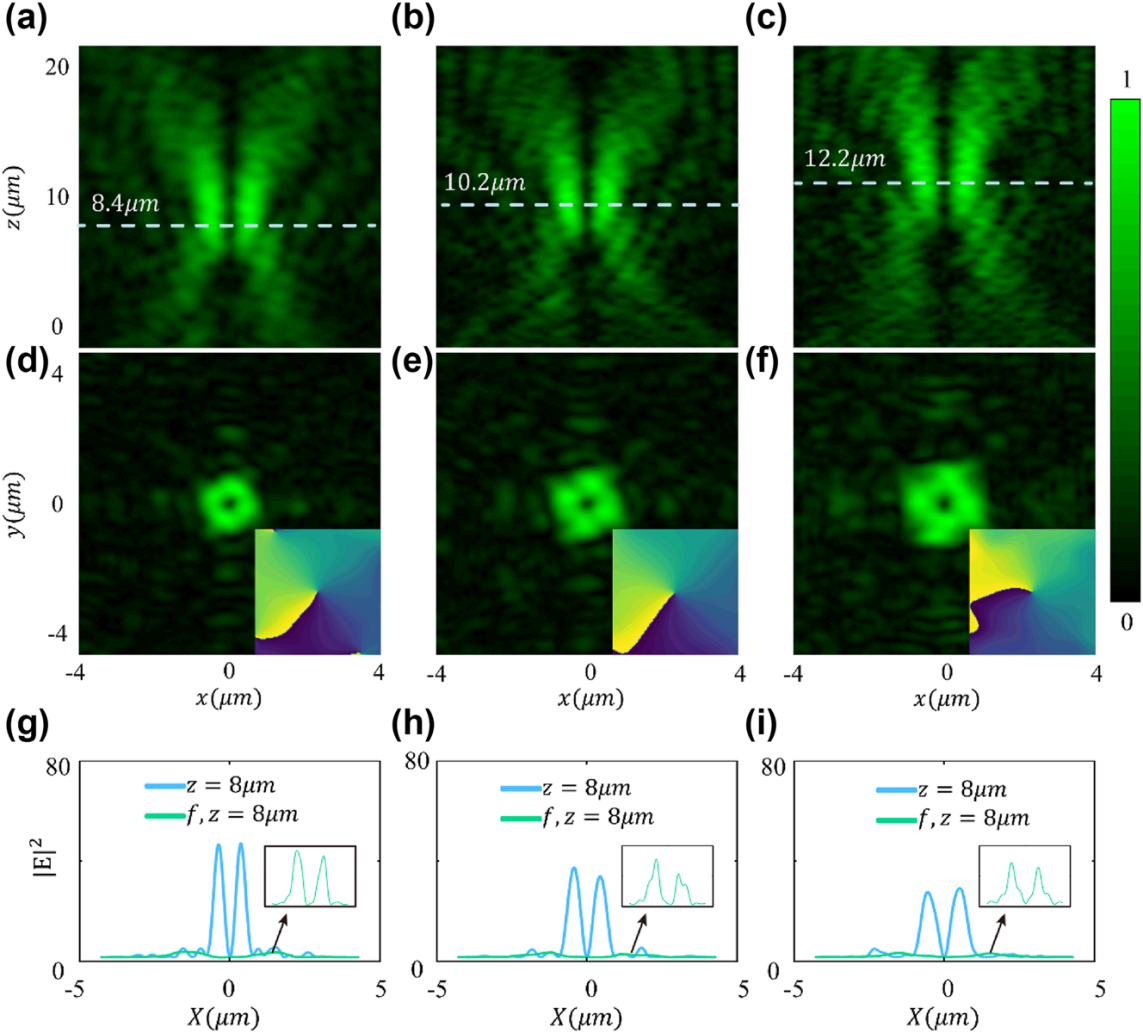
Electric field intensity and phase distribution of the focused vortex metasurface with topological charge *L* = 1. (a–c) y plane electric fields distributions for designed focal lengths of 8 μm, 10 μm, and 12 μm. (d–f) Ring-shaped intensity distribution and spiral phase distribution at the focal plane. (g) Intensity distribution of the vortex focusing metasurface and unfocused vortex light at *z* = 8 μm, (h) *z* = 10 μm, and (i) *z* = 12 μm. The illustration illustrates the intensity distribution of unfocused vortex light.

According to the convolution principle, we superimposed the vortex phase with the focusing phase, obtaining the coding unit distribution pattern of the vortex focusing metasurface. The same coding units exhibit a spiral distribution pattern radiating outward from the center. We performed numerical simulations on the designed 32×32 vortex focusing metasurfaces for three different focal lengths. As shown in Figure 4a–c, under the 532 nm x-polarized light incidence, the focal lengths of the transmitted focused vortices in the x-z plane are 8.4 μm, 10.2 μm, and 12.2 μm, respectively, which are nearly identical to the designed metasurface focal lengths. In the *x* − *y* plane, a “donut”-shaped vortex amplitude and spiral phase are observed, as shown in Figure 4d–f. The hollow circular amplitude and spiral phase curves of the vortex verify that the designed metasurface exhibits good focusing vortex functionality in the transmitted mode.

Figure 4g–i show the intensity comparison of the vortex light before and after focusing at the designed focal lengths, with the same 32×32 array size. Due to the inherent properties of the vortex wave, the light intensity at the precise center of the focus remains almost stable at zero. Additionally, it can be observed that after adding the focusing function, the intensity of the generated vortex light at the same far-field distance increases significantly. At focal lengths of 8 μm, 10 μm, and 12 μm, the electric field intensity ( ${\left|E\right|}^{2}$ ) reaches 42.3, 33.5, and 25.8, respectively, whereas the intensity of the unfocused vortex light at the corresponding *z* = 8 μm, *z* = 10 μm, and *z* = 12 μm planes is only 2.1, 1.7, and 1.6, respectively. This method enhances the energy of the vortex beam near the focus by about 20 times, 19 times, and 16 times. Of course, by enlarging the coding array size, we can theoretically achieve vortex light focusing metasurfaces for larger focal lengths.

Optical vortices have been extensively studied and applied in optical communications. By carrying orbital angular momentum (OAM), optical vortices significantly enhance the communication capacity of optical systems and can be utilized for information modulation and demodulation [41]. Our design, through precise control over vortex beam generation and focusing, directly enhances the scalability and efficiency of OAM-multiplexed communication systems. Furthermore, it supports more complex encoding schemes, thereby improving the performance of secure communication channels [42]. Optical tweezers rely on momentum transfer between light and objects for manipulation. Liu et al. proposed an nth-order alternating optical vortex array with positive and negative topological charges, demonstrating potential applications in particle trapping, advanced optical array experiments, and interactions with optical lattices involving atoms, molecules, ions, or nanoparticles [43]. Our metasurface design enables the generation and control of vortex beams, thereby achieving simultaneous manipulation of multiple particles. This capability facilitates multi-target trapping and rotation, which are crucial for nanoscale assembly or precise quantum manipulation.

## 4 Conclusions

This study, based on the principle of resonant phase, designed high-efficiency transmission units using titanium dioxide nanocylinders on a quartz substrate to construct coding units, and designed a 3 bit coding metasurface with 8 coding units that maintains a consistent broadband phase difference. At the target wavelength of 532 nm, the reflection phase difference between adjacent units remains approximately 45°. Using the above 3 bit coding units, a vortex beam is generated by arranging the 8 units in a spiral pattern into a coding metasurface. At the target wavelength, it can modulate the transmitted light into a vortex wave, with its topological charge varying according to the coding sequence. By combining the principle of Fourier convolution, the vortex beam-coded metasurface sequence is convolved with a gradient phase sequence to obtain a vortex beam with a certain deflection angle. Finally, by combining vortex beams with metasurface lenses, the focused vortex beams achieved an intensity enhancement of 16–20 times at different designed focal lengths. This result validates the vortex beam focusing capability of the metasurfaces, paving the way for new methods of manipulating small particles using vortex waves.

## Supporting information

Supporting information includes the detailed design and simulation results of the gradient sequence metasurface and metalens.

## Supplementary Material

Supplementary Material Details
