# Supplementary material for: Multifunctional metasurface coding for visible vortex beam generation, deflection and focusing
Source: Nanophotonics. 2025 Mar 7;14(5):647–56. doi: 10.1515/nanoph-2025-0016 (PMC11953719; doi:10.1515/nanoph-2025-0016)
Supplement: Supplementary file 1 — Supplementary Material Details [file j_nanoph-2025-0016_suppl_001.docx]

**Supporting Information**

**Multifunctional Metasurface Coding for Visible Vortex Beam Generation, Deflection and Focusing**

**Run Tian, Zhixiao Zhang and Li Gao^*^**

1. **COMPARISON WITH PREVIOUS DESIGNS**

To compare the performance of metasurfaces in generating vortex beams, we introduce the mode purity of multi-mode vortex beams and extract the OAM mode spectrum from the calculated and measured electric field distributions. By sampling the field in the region, the power of different OAM modes can be obtained using Equation (1).

$$\begin{aligned} p_{l}=\frac{1}{2\pi}\int_{0}^{\infty} \left| \int_{0}^{2\pi} u\left( x,y \right)\cdot\exp\left( -jl\varphi\right)d\varphi\right|^{2}rdr\#\left( 1 \right) \end{aligned}$$

Where $u(x, y)$ represents the complex amplitude of the electric field. Additionally, since the power of modes with $|l| > 5$ is very weak, we only calculate the OAM mode spectrum for $l\in[-5,5]$. Then, we using Formula 2 to calculate the purity ratio of the target mode.

$$\begin{aligned} P_{l}=\frac{p_{l}}{\sum_{q=-\infty}^{+\infty} p_{q}}\#\left( 2 \right) \end{aligned}$$

$P_{l}$represents purity, which is the proportion of the target mode in the generated vortex beam modes. Then, we can calculate the vortex beam conversion efficiency using the formula $\eta=PT$, where $T$ represents the transmission of vortex.

**Table S1.** Comparison of Metasurface Vortex Beam Purity

| Ref. | [22] | [38] | [39] | [40] | This work |
| --- | --- | --- | --- | --- | --- |
| $\lambda$ | 532nm | 20mm | 16.7mm | 29.6mm | 532nm |
| Transmission/Reflection($l=1$) | 83% | 98% | 78% | 94% | 92.28% |
| Transmission/Reflection($l=2$) | 67.9% | 98% | 75% | 94% | 92.83% |
| $P_{1}$ | 95.6% | 77.4% | 75% | 77.2% | 81.45% |
| $P_{2}$ | 83.9% | 66.1% | 80% | 81.8% | 82.83% |
| $\eta_{1}$ | 79.35% | 75.85% | 58.5% | 74.88% | 75.16% |
| $\eta_{2}$ | 56.97% | 64.78% | 60% | 76.89% | 76.89% |
| Multifunctional integration | No | Focus | Deflection | No | Focus/  Deflection |

Taking recent works [22], [38], [39], and [40] as examples, we compared the purity of vortex beams generated by metasurfaces, as shown in Table S1. For Reference [22], the complex hollow nanocylinder structure employed achieved higher purity vortex beams through deep learning optimization of more parameters. However, the high aspect ratio combined with the complex structure resulted in relatively low transmission, leading to suboptimal conversion efficiency. In contrast, our nanocylinder design reduces system complexity and computational costs while maintaining good purity, high transmission, and conversion efficiency. Additionally, it enables multifunctional integration through the convolution theorem, thereby providing relative advantages in practical engineering applications.

1. **BROADBAND SIMULATION RESULTS**

To explore the applicability of this design over a broader spectral range, we conducted simulations for wavelengths near the operating wavelength and selected several representative wavelengths for analysis, as shown in Table S2. The results indicate that the vortex beam purity for $l=1$ and $l=2$ decreases, suggesting that the design maintains acceptable performance within a narrower spectral range.

Regarding broadband design, our metasurface relies on mode resonance within the unit structure, and complete $2\pi$ phase control can only be achieved near the resonance frequency, which imposes certain limitations on the operational bandwidth. To address this issue, we can optimize the unit structure parameters to not only provide $2\pi$ phase coverage but also exhibit anomalous dispersion characteristics. By introducing a reference phase $C$ and employing particle swarm optimization (PSO) to minimize the phase deviation between the designed and required phase across different wavelengths, it is promising to achieve phase overlap over multiple wavelengths, thereby enhancing broadband performance.

**Table S2.** Vortex Beam Purity at Different Wavelengths

| $\lambda$ | 580 | 550 | 532 | 500 |
| --- | --- | --- | --- | --- |
| $P_{1}$ | 76.82% | 79.64% | 81.45% | 80.69% |
| $P_{2}$ | 71.53% | 78.26% | 82.83% | 68.30% |

1. **ROBUST ANALYSIS FOR THE METASURFACE**

Surface roughness and fabrication deviations typically affect device performance. Considering that high-quality electron beam lithography and reactive ion etching can achieve fabrication deviations below 10 nm in experiments, we simulated realistic deviations by uniformly increasing and decreasing the nanocylinder radius by 10 nm. The simulation results, as shown in Table S3, indicate that the vortex beam purity decreases by less than 10%, with a smaller decline for vortex beam with $l=1$. This demonstrates that the designed metasurface exhibits good robustness during the fabrication process.

**Table S3.** The robust analysis for the metasurface

| Purity | Error free | +10 nm error | -10 nm error |
| --- | --- | --- | --- |
| $P_{1}$ | 81.45% | 79.34% | 79.58% |
| $P_{2}$ | 82.83% | 74.12% | 74.43% |

1. **GRADIENT METASURFACE DESIGN**

According to the angular addition calculation rule, the two encoding sequences are designed to obtain the coding metasurface sequence that can deflect the vortex beam. We first designed a $16\times16$ phase gradient sequence (01234567) and performed numerical simulations of the far-field scattering of the designed phase gradient sequence using the finite-difference time-domain (FDTD) method, as shown in Figure S4(a). We also designed a $32\times32$ array with a $2\times2$ repeating unit using the same encoding, and its far-field simulation pattern is shown in Figure S4(b). Figure S4(c) presents the normalized far-field intensity patterns of the two arrays, where we can intuitively observe that the corresponding deflection angles $\theta_{1}$ and $\theta_{2}$ are $15.8^{\circ}$ and $7.8^{\circ}$, respectively. Comparing these values with the theoretical values obtained from Equation (3), we find that the numerical simulation results are in good agreement with the theoretical results. Meanwhile, by repeating the design of encoding units, we can achieve different beam deflection angles.


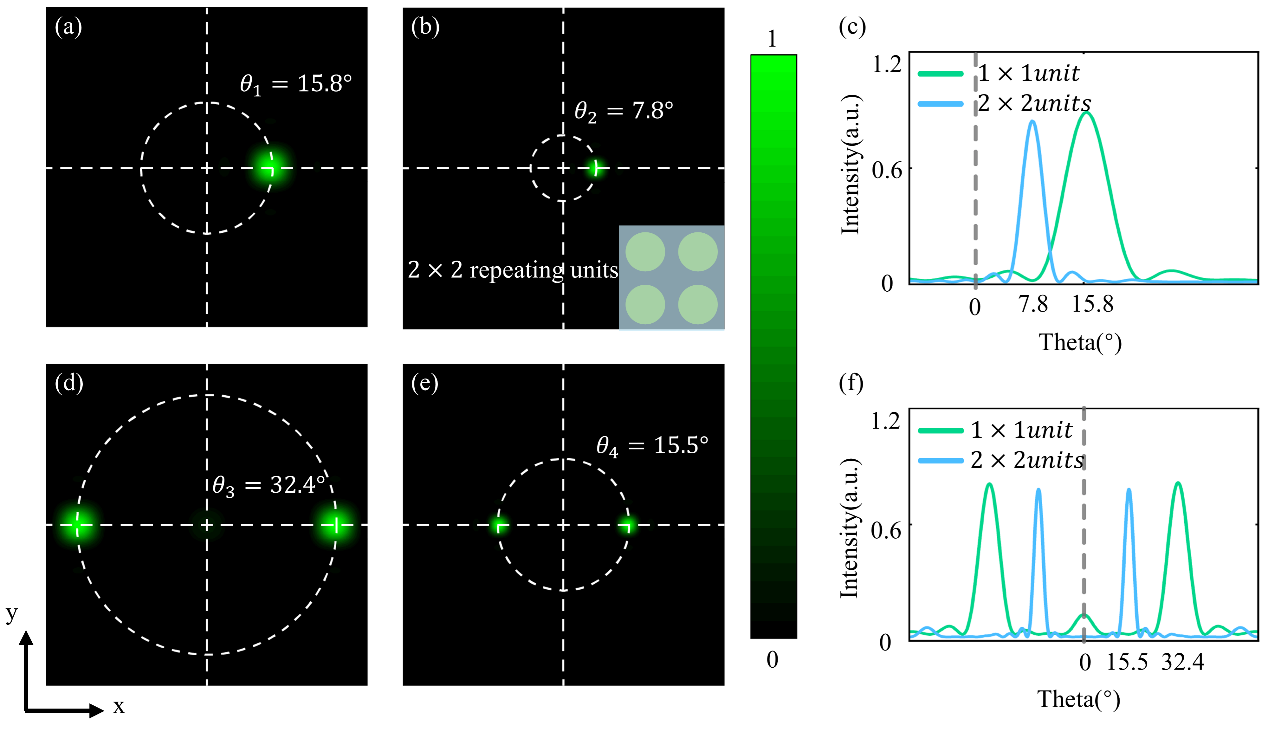


**Figure S4:** Beam deflection angle control. (a) Far-field Intensity Distribution in the Z-plane of a $16\times16$ “01234567” Gradient Sequence, the Radius of the White Dashed Circle Representing the Deflection Angle. (b) Far-field Intensity Distribution of the Array After $2\times2$ Repetition of the Coding Units. (c) Normalized Far-field Intensity Distribution of two “01234567” Arrays and Corresponding Deflection Angles are 15.8°and 7.8°.(d) Far-field Intensity Distribution in the Z-plane of a $16\times16$ “73377337” Gradient Sequence, with the Transmitted Beam Divided into Two Symmetric Beams. (e) Far-field Intensity Distribution of the Array After $2\times2$ Repetition of the Coding Units. (f) Normalized Far-field Intensity Distribution of the Two “73377337” Gradient Sequence Metasurfaces and Corresponding Beam Deflection Angles are 32.4° and 15.5°.

Next, based on the principle of complex digital field encoding addition, we obtained the phase gradient sequence for dual-lobe deflection (73377337). We similarly designed two arrays and performed simulations to obtain the far-field patterns shown in Figure S4(d) and 4(e). It can be observed that the emitted beam is divided into two lobes. Figure S4(f) shows the far-field intensity maps of the two arrays, where the deflection angles $\theta_{3}$ and $\theta_{4}$ are $32.4^{\circ}$ and $15.5^{\circ}$, respectively, which are in good agreement with the theoretical results. In far-field scattering, besides the main scattering lobe, there are also some side lobes. These may arise due to the interactions between unit structures. We designed the phase values of the unit structures, and when these unit structures are arranged, they may change due to interactions between particles, leading to the formation of side lobes. These side lobes can be reduced by increasing the number of units on the metasurface or by using global optimization methods.

1. **METALENS DESIGN**

**
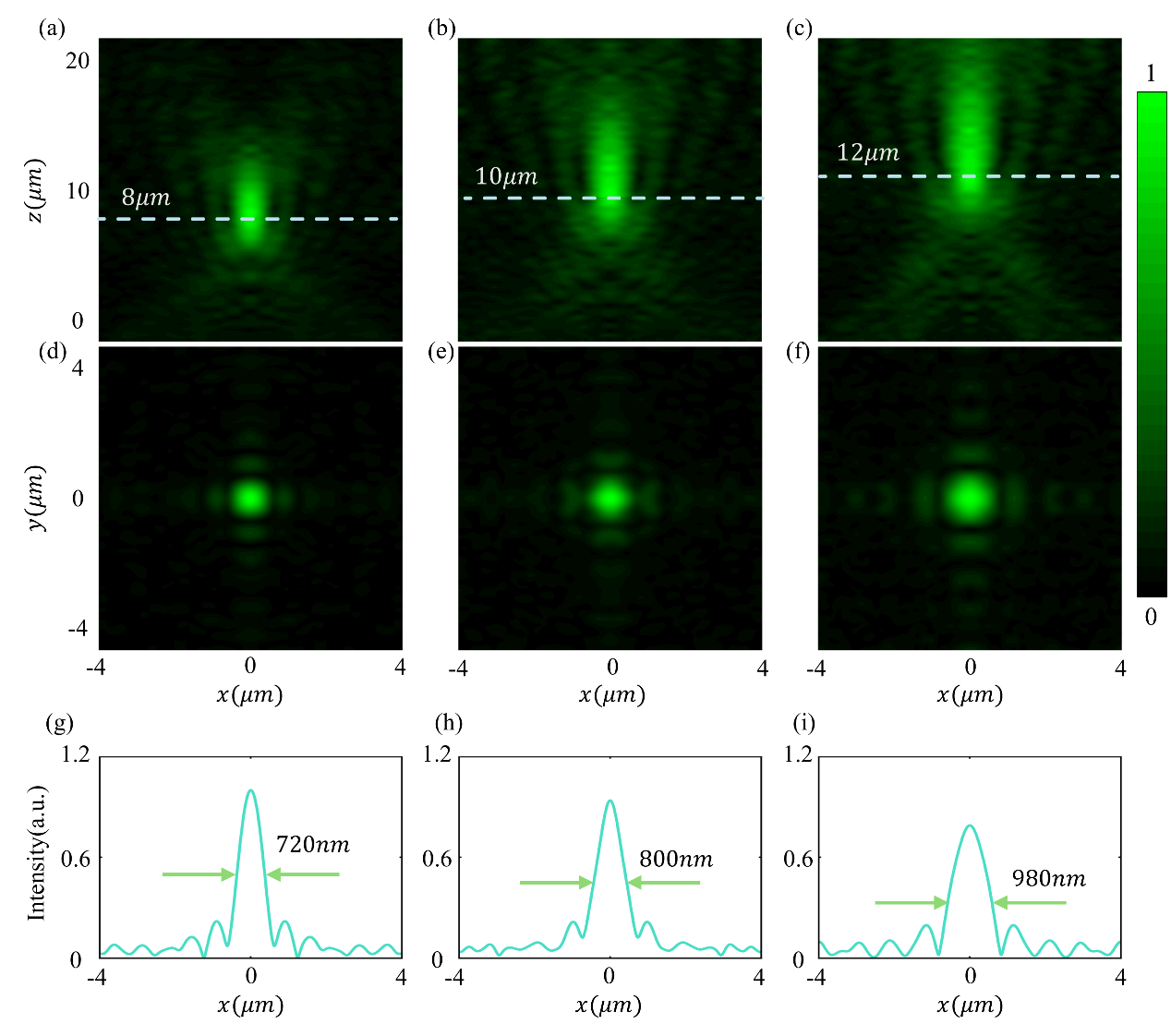
**

**Figure S5: Metasurface Lens Design** (a-c) Simulated transmitted electric field of the designed 32x32 encoded metasurface array at different focal lengths ($8 \mu m$, $10 \mu m$, $12 \mu m$). The white dashed line indicates the position of the focal plane. (d-f) Intensity distribution of three different metasurfaces with designed focal lengths in their respective focal planes. (g-i) Normalized intensity distribution along the horizontal (y-axis) direction passing through the center of the focal point and the corresponding focal spot sizes.

To demonstrate the focusing performance of the designed metasurfaces, simulations were conducted for the three focal length designs. Under the incident x-polarized light, the transmitted electric fields are shown in **Figure S5(a-c)**. As the focal length increases, the focusing performance deteriorates, possibly due to phase dispersion causing a non-ideal phase distribution (deviating from the ideal value designed in Equation (6)). The electric field maps in the focal plane for different focal lengths are shown in **Figure S5(d-f)**. **Figure S5(g-i)** show the normalized power flow intensity along the horizontal (y-axis) line passing through the focal point and the corresponding focal spot sizes for different focal lengths. The focal spot size reflects the metasurface's ability to converge spatial waves (the smaller the spot size, the stronger the convergence ability)
